# The scholar’s best friend: research trends in dog cognitive and behavioral studies

**DOI:** 10.1007/s10071-020-01448-2

**Published:** 2020-11-21

**Authors:** Massimo Aria, Alessandra Alterisio, Anna Scandurra, Claudia Pinelli, Biagio D’Aniello

**Affiliations:** 1 Department of Economics and Statistics, University of Naples Federico II, via Cinthia, 80126 Naples, Italy; 2 Department of Biology, University of Naples Federico II, via Cinthia, 80126 Naples, Italy; 3 Department of Environmental, Biological and Pharmaceutical Sciences and Technologies, University of Campania “Luigi Vanvitelli”, Caserta, Italy

**Keywords:** Dog, Bibliometrix, Behavioral science, Science mapping, Cognition, Behavior

## Abstract

In recent decades, cognitive and behavioral knowledge in dogs seems to have developed considerably, as deduced from the published peer-reviewed articles. However, to date, the worldwide trend of scientific research on dog cognition and behavior has never been explored using a bibliometric approach, while the evaluation of scientific research has increasingly become important in recent years. In this review, we compared the publication trend of the articles in the last 34 years on dogs’ cognitive and behavioral science with those in the general category “Behavioral Science”. We found that, after 2005, there has been a sharp increase in scientific publications on dogs. Therefore, the year 2005 has been used as “starting point” to perform an in-depth bibliometric analysis of the scientific activity in dog cognitive and behavioral studies. The period between 2006 and 2018 is taken as the study period, and a backward analysis was also carried out. The data analysis was performed using “bibliometrix”, a new R-tool used for comprehensive science mapping analysis. We analyzed all information related to sources, countries, affiliations, co-occurrence network, thematic maps, collaboration network, and world map. The results scientifically support the common perception that dogs are attracting the interest of scholars much more now than before and more than the general trend in cognitive and behavioral studies. Both, the changes in research themes and new research themes, contributed to the increase in the scientific production on the cognitive and behavioral aspects of dogs. Our investigation may benefit the researchers interested in the field of cognitive and behavioral science in dogs, thus favoring future research work and promoting interdisciplinary collaborations.

**Electronic supplementary material:**

The online version of this article (10.1007/s10071-020-01448-2) contains supplementary material, which is available to authorized users.

## Introduction

The domestication of wolves was probably the first human successful attempt aimed to control an animal. After the first stage, in which primitive dogs were domesticated from their wild ancestors—the wolves, dogs were artificially selected resulting into the modern breeds, based on different specialization and morphology (Wayne and Ostrander 2007). However, several aspects of this history remain unclear, despite scientific efforts in studying dog evolution. Under dispute is the place of the domestication process of dogs—from Europe or southern East Asia—as uncertainly lies in the divergence between wolves and dogs (Thalmann et al. 2013; Wang et al. 2016). Many doubts also surround the question of how domestication of dogs began and how this process has impacted cognition and behavior in dogs (Hare et al. 2002; Udell and Wynne 2008; Wynne et al. 2008; Topál et al. 2009a). Probably, the secret of such a long duration and effective cooperation with humans are dogs’ advanced social skills, which allowed them to exchange communicative signals effectively with the human (Miklósi 2009; D’Aniello and Scandurra 2016; D’Aniello et al. 2017; Scandurra et al. 2017, 2018).

In history, dogs were mostly employed for utility, whereby the term “man’s best friend” originated in the eighteenth century (see Miklósi and Topál 2013). They are now increasingly involved in different working and sporting activities, and their presence in our homes as pets is also in a growing trend in many countries (see, for example, Murray et al. 2015). Pet dogs could promote the welfare of the human family they live with, whereas working dogs are an integral part of social functioning. Several studies demonstrated that keeping dogs has a positive effect on our physical and mental health (Levine et al. 2013; Ownby et al. 2002; Raina et al. 1999; Kramer et al. 2019). Although this so-called “pet effect” (Allen 2003) received some criticism, noting that some papers reported null or also negative effects on health and happiness of pet owners (see Herzog 2011 for a review), it contributed to the flurry of research on dogs.

Altogether, dogs have become an important social phenomenon attracting scientific interest (Morell 2009). There are different reasons why canine research is advantageous beyond the easy access to the subjects for experimental purposes. They show many similarities with humans (Scandurra et al. 2020) and, therefore, can be used as a model for human studies. Indeed, there are functional parallels in a range of behavioral features, which are not shared with the closest human relatives, the great apes (Topál et al. 2009b). The success of dogs as behavioral models also relies on their origin from ancestors with high social behavior, the adaptiveness in living in the anthropogenic niches and the socialization with humans during ontogeny (Kubinyi et al. 2009). Moreover, dogs have also been used as a model for comparative and translational neuroscience, cancer and cognitive decline, such as in Alzheimer’s disease in humans (Head et al. 2000).

Bibliometric research focusing specifically on the dog’s personality or temperament showed about 50 papers in the database between 1934 and 2004 (Jones and Gosling 2005). A comprehensive study by Bensky et al. (2013) aimed at identifying the major trends in the literature related to the areas of cognitive research on dogs, detected an increase in the studies over the 15 years before the date of the review. However, a worldwide trend of scientific research on dog cognition and behavior has never been explored to date using a bibliometric approach (Chen 2003), while the evaluation of scientific research has increasingly become important in recent years. Bibliometric analysis is a useful tool to measure the output of scientific research, using specific indicators to obtain information about the research trends in different fields (De Battisti and Salini 2013; Wallin 2005).

There are pure cognitive studies, which analyze the brain functioning through brain imaging (e.g., fMRI studies), without taking into account the behavioral responses. However, most of the papers dealing with cognition also include behavioral outcomes. Indeed, brain imaging studies often analyze brain functioning, while the experimental subjects are performing various behavioral tasks. Moreover, many studies provide data for the understanding how stimuli are processed (i.e., cognition) by studying behavioral responses. Therefore, it is not so common to find pure cognitive or behavioral studies, whereby, in this paper, we have considered all studies dealing with both cognition and/or behavior.

Since there is evidence of a growing trend in scientific production (Fanelli and Larivière 2016), the first goal of the present paper was to verify whether the dog cognitive and behavioral studies show a growing trend exceeding those of cognitive and behavioral sciences in general. To this scope, we have provided a comparison between the trend in the literature on dogs and that of the whole collection of studies in the subject category “Behavioral Sciences” from 1985 (i.e., the year of starting electronic access to Web of Science database) to 2018. It was verified that peer-reviewed publications on dog cognitive and behavioral studies showed a steeper growth curve with respect to that of the subject category “Behavioral Sciences” starting from 2005. Therefore, this year was chosen as a “starting point” for a “recent analysis” until 2018, including 13 years. To further emphasize the more current changes in the scientific production related to the cognitive and behavioral studies on dogs, we compared the “recent analysis” with an “earlier analysis”. The latter covered in the backward direction an equivalent number of years to the “recent analysis” from the “starting point”.

Our second goal was to understand whether the growth of scientific production in dog cognition and behavior was simply related to an increased research effort in the same research themes or changes in research themes and the contribution of new research themes to this trend.

The further aim was to provide a bibliometric analysis related to sources, countries, affiliations, co-occurrence network, thematic maps, collaboration network, and world maps of the scientific activity related to the cognitive and behavioral studies on dogs. We also attempted to identify the most frequent and impactful journals, countries, research institutes, and their relationship at social and conceptual levels.

Overall the information reported in this study could be useful to the researchers in locating the topics that need more scientific efforts, giving information to help further develop the already thriving growing field of dog cognition and behavior, thus fostering future interdisciplinary collaborations.

We used bibliometrix, a new R-tool for comprehensive science mapping analysis (Aria and Cuccurullo 2017), which provides various options for importing bibliographic data from scientific databases and performing bibliometrics analysis related to different items. We employed bibliometrix to analyze the sources, countries, and affiliations. It allowed us to define the structure of the topic at the conceptual level based on the co-occurrence network and thematic maps and social structure as gathered by collaboration network and world maps.

## Methods

### Selection strategy

Our investigation followed the Preferred Reporting Items for Systematic Reviews and Meta-Analysis (PRISMA) guidelines, illustrating the outcomes of the literature searches and article selection process (Liberati et al. 2009). PRISMA consists of a checklist describing the protocol adopted for selecting the collection of articles used in a systematic literature review. It is used to ensure that the selection process is replicable and transparent. We performed a computerized bibliometric analysis from January 1985 to December 2018 for articles retrieved from the Web of Science (WoS) database, which is now maintained by Clarivate Analytics, and also retrieved articles from the Science Citation Index Expanded (SCI expanded) and the Social Science Citation Index (SSCI). Data were collected in January 2020.

To identify all publications related to this field, we defined the following query: (((TS = (((dog OR dogs) AND *cogniti*) OR (canis AND familiaris AND *cogniti*))) OR (TS = ((( dog OR dogs) AND communicat*) OR (canis AND familiaris AND communicat*))) OR (TS = ((( dog OR dogs) AND behav*) OR (canis AND familiaris AND behav*))))). TS stands for topic, that is, the search of the mentioned words in the title, abstract, and keyword lists. This query was formulated after some exploratory trials in which, after using the word behav* and cogniti*, we noted that some of the publications in our personal database related to dogs’ communication did not appear. Thus, for a more comprehensive research, we also added the word communicat*. In our search, we selected original articles in the English language, including experiments (i.e., review articles and proceedings were excluded).

The information about the retrieved articles by WoS in Bib TeX format was exported into Microsoft Excel 2017. The selection involved two selectors, which reached a satisfactory agreement level (Cohen’s *K* = 0.91). The choices that did not match were resolved involving a third independent researcher and the final decision was taken by the concensus among researchers (Cuccurullo et al. 2016).

The inclusion criteria concerning cognitive and behavioral sciences are reported in Table 1.

**Table 1:** Research considered for the selection

FMRi studies
Lateralization
Memory
Perception
Attention
Learning
Spatial cognition
Numerical abilities
Reasoning and problem solving
Training and working dogs (including assistant and therapy dogs, unless the target is the human benefit)
Drug treatments (in the case untreated controls are involved; e.g., oxytocin)
Aging (as a natural cognitive decline of behavioral outcomes)
Theory of mind
Welfare and stress in general
Emotions
Temperament and personality
Studies with questionnaires (unless aimed to study behavioral problems)
Behavioral ecology (predation, scavenger behavior etc.)
Domestication (when they deal with cognitive evolution)

The papers were enclosed in our collection when behavioral responses or brain processing related to cognition is considered

### Data loading and converting

Numerous software tools support science mapping analysis; however, many of these do not assist scholars in a complete recommended workflow. The most relevant tools are bibliometrix (Aria and Cuccurullo 2017), CitNetExplorer (van Eck and Waltman 2014), VOSviewer (van Eck and Waltman 2010), SciMAT (Cobo et al. 2012), and CiteSpace (Chen 2006). Starting from our final collection, we loaded the data (i.e., the selected papers matching the inclusion criteria, including all their metadata) and converted it into R data frame using bibliometrix (Aria and Cuccurullo 2017) since it contains a more extensive set of techniques and it is suitable for practitioners through Biblioshiny (Moral-Muñoz et al. 2020).

To investigate the interest in dog’s cognitive and behavioral sciences, the annual trend of publications from 1985 to 2018 was compared with the whole literature in the field of cognitive and behavioral studies published in the subject category “Behavioral Science,” since most of the papers related to cognition and behavior fall in this subject category. Indeed, it includes 53 journals, some of them with the main focus on cognition, such as “*Animal Cognition*” and other focusing mainly on behavior, such as “*Animal Behavior*”. However, all journals in the subject category “Behavioral Science” tend to accept studies on cognition and/or behavior.

From 2005, the studies on dogs diverged upward from the general growing trend of the papers on cognitive and behavioral studies published in the subject category “Behavioral Science” (see results). Thus, we chose to use this point to perform the following separate analysis on dogs a posteriori. A “recent analysis”, including the last 13 years (2006–2018), was used to underline emerging aspects, and an “earlier analysis”, counting the same number of years (1993–2005), was used for comparative purposes. In this way, we were able to compare the period in which there was an increase in the scientific production on dogs exceeding the trend of studies in the subject category “Behavioral Sciences” and an equivalent number of years in which the growing trend of dogs’ cognitive and behavioral studies paralleled that of behavioral sciences. This choice allowed us to test whether the exceeding trend of dog studies was simply due to an increased effort in the same topics or new topics contributed to this increase.

We analyzed the article collection using different aggregation levels. Regarding journals, bibliometrix provides many indicators, such as the number of publications, h-index (Hirsch 2005), g-index (Egghe 2006), m-index (von Bohlen und Halbach 2011), and the total number of citations. Thus, we reduced the variables by applying a principal component analysis with orthomax rotation, through a statistical tool for Excel (XLSTAT 2019, Addinsoft Inc.).

Co-occurrence network, collaboration network, thematic maps, and world maps are also provided. A network is a graphical representation of item co-occurrences in a set of documents. In a co-occurrence network, the items consist of terms extracted from the article keyword lists, from the titles, or from the abstracts; while in a collaboration network, the items consist of the co-authors, the author’s affiliations, or the author’s countries. A thematic map is a Cartesian representation of the term clusters identified performing a cluster analysis on a co-occurrence network. It allows for easier interpretation of the research themes developed in a framework. Finally, a world map is a geographical representation of the collaboration network of an author’s country. The analyses were based on KeyWords Plus, which are the words or phrases that frequently appear in the titles of the references cited in an article but do not appear in the title of the article itself. They are extracted from the papers using a statistical algorithm, based on the cited references in the article. This process is unique to Clarivate Analytics databases. The algorithm is based on a supervised machine learning approach that automatically assigns a set of keywords, namely, Keyword Plus, from a glossary defined by a team of experts. This approach uses the article’s bibliography to identify the research topics and then label the document with a set of Keyword Plus. The use of the KeyWords Plus offers several advantages over other databases and author’s keyword list, in such a way that the terms are extracted from a standardized glossary, defined for subject categories analyzed. It also covers a larger knowledge base and unbiased concerning the author’s subjectivity when providing keywords for their articles (Zhang et al. 2016). Moreover, a comparison between Keywords Plus and Author Keywords performed at the scientific and the document levels yields more Keywords Plus terms than Author Keywords, and it is more descriptive (Zhang et al. 2016).

Based on Keywords Plus, we obtained the co-occurrence network, which identifies the relationship between the keywords. Each keyword represents a node, or vertex, of the network, and the edge connecting two nodes is proportional to the number of times two keywords are included in the same keyword list. Stronger is an edge, higher is the relationship between two keywords within a paper (Tijssen and Van Raan 1994), thus allowing to provide a graphic visualization of potential relationships among keywords. In the network, it is possible to identify groups of strongly interconnected terms, which represent themes or topics. Although different algorithms exist to identify these groups, this study used the Louvain community detection algorithm (Blondel et al. 2008) because it gave the best results when applied to different benchmarks on Community Detection methods (Lancichinetti and Fortunato 2009).

The clusters identified by the co-occurrence network were plotted in a thematic map according to Callon’s centrality and Callon’s density rank values along the two axes (Callon et al. 1991).

The X-axis represents the centrality, that is, the degree of interaction of a network cluster in comparison with other clusters appearing in the same graph. It can be read as a measure of the importance of a theme in the development of the research field. The Y-axis symbolizes the density, which measures the internal strength of a cluster network, and it can be assumed as a measure of the theme’s development (Cahlik 2000; Cobo et al. 2011, 2015). According to these authors, the graphical representation of themes on the four quadrants in which they are plotted allows identification of the following proprieties: (1) *Motor themes* (first quadrant): the cluster network is characterized by high centrality and high density, meaning that they are well developed and important for the structuring of a research field; (2) *Highly developed and isolated themes* (second quadrant): they are characterized by high density and low centrality, meaning that they are of limited importance for the field since they do not share important external links with other themes; (3) *Emerging or declining themes* (third quadrant): they have low centrality and low density, meaning that they are weakly developed and marginal. The identification of emerging or declining trends of a theme requires a longitudinal analysis, through a thematic evolution (Aria and Cuccurullo 2017): splitting the timespan into different timeslices allow to identify the trajectory, whereby a direction toward the top of the map over time identifies an emerging trend while a direction toward the lower left quadrant would identify a declining trend; (4) *Basic and transversal themes* (fourth quadrant): they are characterized by high centrality and low density, namely, they are important concerning general topics that are transversal to different research areas of the field.

The scientific collaboration analysis was used to identify the social structure of the field, through the application of the social network analysis (Newman 2001), applying it at an aggregated level (i.e., countries).

## Results

**Fig. 1:**
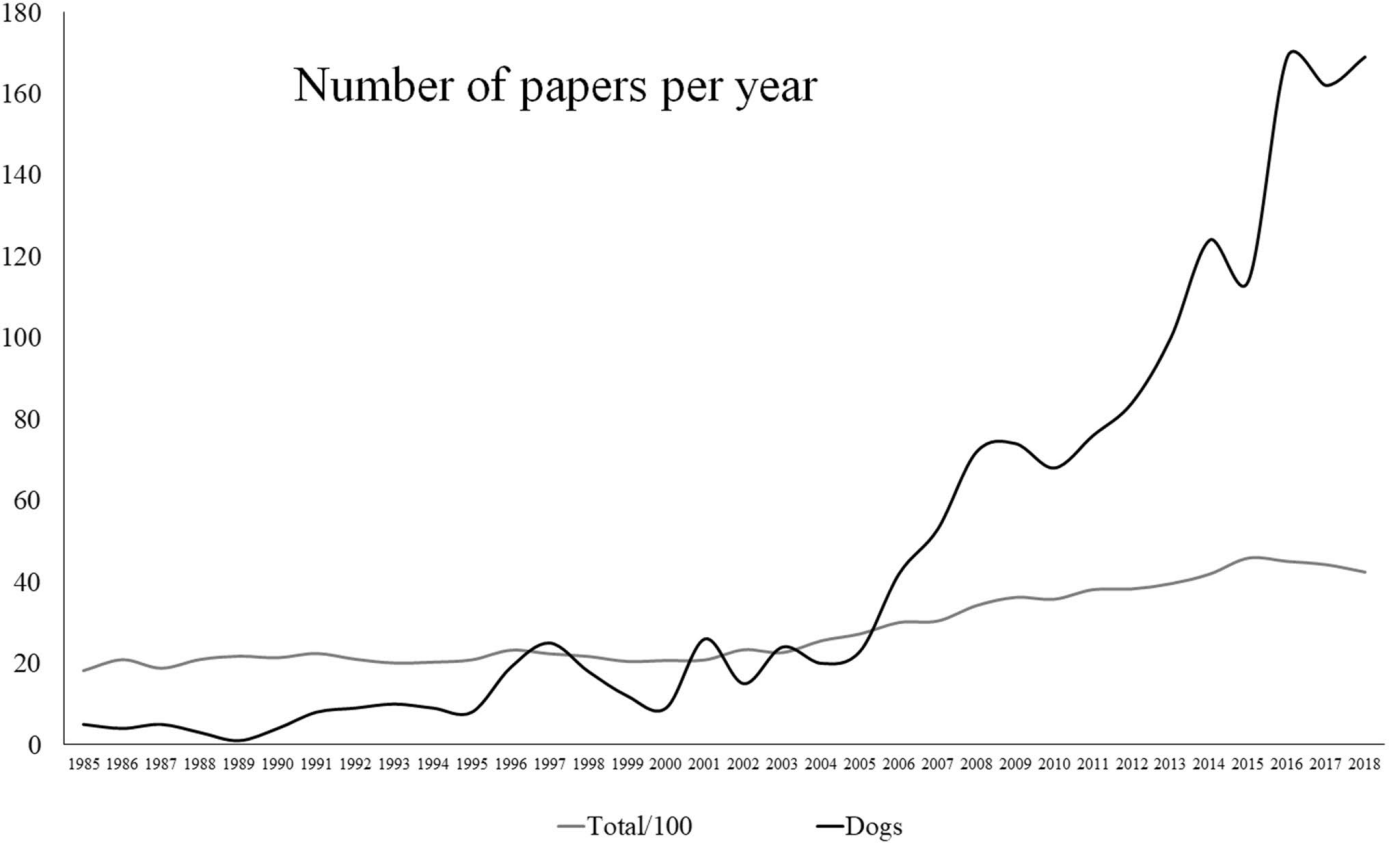
Comparative view of the annual scientific production related to cognitive studies on dogs (black line) and the trend of publication rate of research in the subject category “Behavioral Sciences” (gray line; for an obvious comparison, the line has been lowered 100 folds). It is evident that increasing trend in research on dogs starts from 2005

The comparison of the number of publications from selected papers for dogs with the general trend of all papers published in the subject category “Behavioral Science” showed that starting from 2005, there has been a sharp increase in scientific production on dogs (Fig. 1).

Data related to the main information on dogs are reported in Table 2.

**Table 2:** Main information about the collection reported separately for “earlier analysis” (EA: 1993–2005) and “recent analysis” (RA: 2006–2018), with EA-RA variation

Main information about collection			
Period	EA (1993–2005)	RA (2006–2018)	EA-RA variation
Documents	218	1307	5.00
Sources	34	85	1.50
Keywords plus (ID)	493	2557	4.19
Author’s keywords (DE)	368	2462	5.69
Average citations per documents	62	15	− 0.76
Authors	397	2666	5.72
Author appearances	722	5393	6.47
Authors of single-authored documents	26	37	0.42
Authors of multi-authored documents	371	2692	6.26
Single-authored documents	34	46	0.35
Documents per Author	0.549	0.490	− 0.11
Authors per document	1.82	2	0.12
Co-Authors per documents	3.31	4	0.25
Collaboration Index	2.02	2	0.03

After our selection, 218 papers related to studies on dogs were retrieved in the “earlier analysis” (1993–2005), while they were 1307 in the “recent analysis” (2006–2018). This means that the scientific production on dog cognitive and behavioral studies increased sixfold. A similar finding was also observed for the Keywords Plus, Authors’ Keywords, Authors, Author Appearances, Authors of multi-authored documents, and single-authored documents. However, these data support the view that there is a considerable increase in the researchers working on the cognitive and behavioral aspects of dogs. It also appears that the contribution of a single researcher who co-authored remains almost unchanged, which means that the research effort by each researcher has not generally increased over time.

### Sources impact

Dog cognitive and behavioral studies appeared in 34 different sources in “earlier analysis”, while they substantially increased in “recent analysis”, totaling to 85. The principal component analysis (PCA), carried out on the number of publications, h-index, g-index, m-index, and the total number of citations, highlighted a single principal component explaining 98.56% of the variability (Eigenvalue = 4.928, *χ*^2^ = 584.479, *P* < 0.001), with *KMO* = 0.786 ensuring the sampling adequacy in the “earlier analysis”. In the “recent analysis”, the PCA detected a single component explaining most of the variability (93.709%, Eigenvalue = 4.685, *χ*^2^ = 875.361, *P* < 0.001,* KMO =* 0.838).

The highest score for the cognitive and behavioral sciences of dogs was the *Applied Animal Behavior Science*, both in “earlier analysis” and “recent analysis”. *Journal of the American Veterinary Medical Association*, occupying the second place in “earlier analysis”, was less utilized in “recent analysis”, not appearing in the first ten sources anymore. A lowering in score from “earlier analysis” to “recent analysis” was also observed for the third journal in the list, which was the *Journal of Comparative Psychology*. In “recent analysis”, *Animal Cognition* and *Journal of Veterinary Behavior-Clinical Applications and Research* both showed an increase in the score, occupying the second and third place, respectively. It is noteworthy that *PLoS One* acquired a high score in dog cognitive and behavioral studies in “recent analysis”. Indeed, it was not present in “earlier analysis” since it was launched in 2006, which coincides with the start of our “earlier analysis” analysis. Similar reasoning could be applied for *Scientific Reports*, which was launched in 2011 and is in the first ten sources in our collection related to the “recent analysis”. Full data for the sources are reported in Online Resources 1 and 2.

### Country productivity and affiliations

According to our collection of the metadata, in the “earlier analysis”, 22 countries contributed to dog cognitive and behavioral studies, whereas in the “recent analysis”, they have almost been doubled (42).

Considering the number of publications related to corresponding authors, the leading countries were the USA, the UK, and Hungary in the “earlier analysis”, and these were also among the most productive in “recent analysis”. Ireland appeared as the fifth most productive country in “earlier analysis” but was much less involved in dog cognitive and behavioral studies in “recent analysis”, where it ranked at the 24th position. A slightly less engagement on the part of the Netherlands affected its ranking, pushing it down from the ten to the fifteenth position. On the contrary, Italy, contributed less in “earlier analysis”, while it appeared more productive in “recent analysis”, ranking in the third position. A similar observation holds for Austria, which was not listed in “earlier analysis”, but appeared in the first ten most productive countries in the “recent analysis”. Japan was not in the top ten contributing countries in “earlier analysis” but was indeed listed in “recent analysis”. However, this country has only earned two positions, thus maintaining its almost unchanged status in terms of its contribution. The whole data of countries’ productivity are given in Online Resources 3 and 4.

Concerning the affiliations, the Eötvös Loránd University of Hungary provided the highest contribution to the development of the dog cognitive and behavioral sciences in the “earlier analysis”, followed by the University of Toronto and the Utrecht University (the Netherlands). This ranking substantially changed in the “recent analysis”. Besides Eötvös Loránd University of Hungary, which has always been the major contributor to developing dog cognitive and behavioral sciences, all affiliations in the top ten were new, with the University of Vienna and the University of Milan occupying the second and third place, respectively, in the list. The list of the most productive affiliations can be found in Online Resources 5 and 6.

### Conceptual structure

**Fig. 2:**
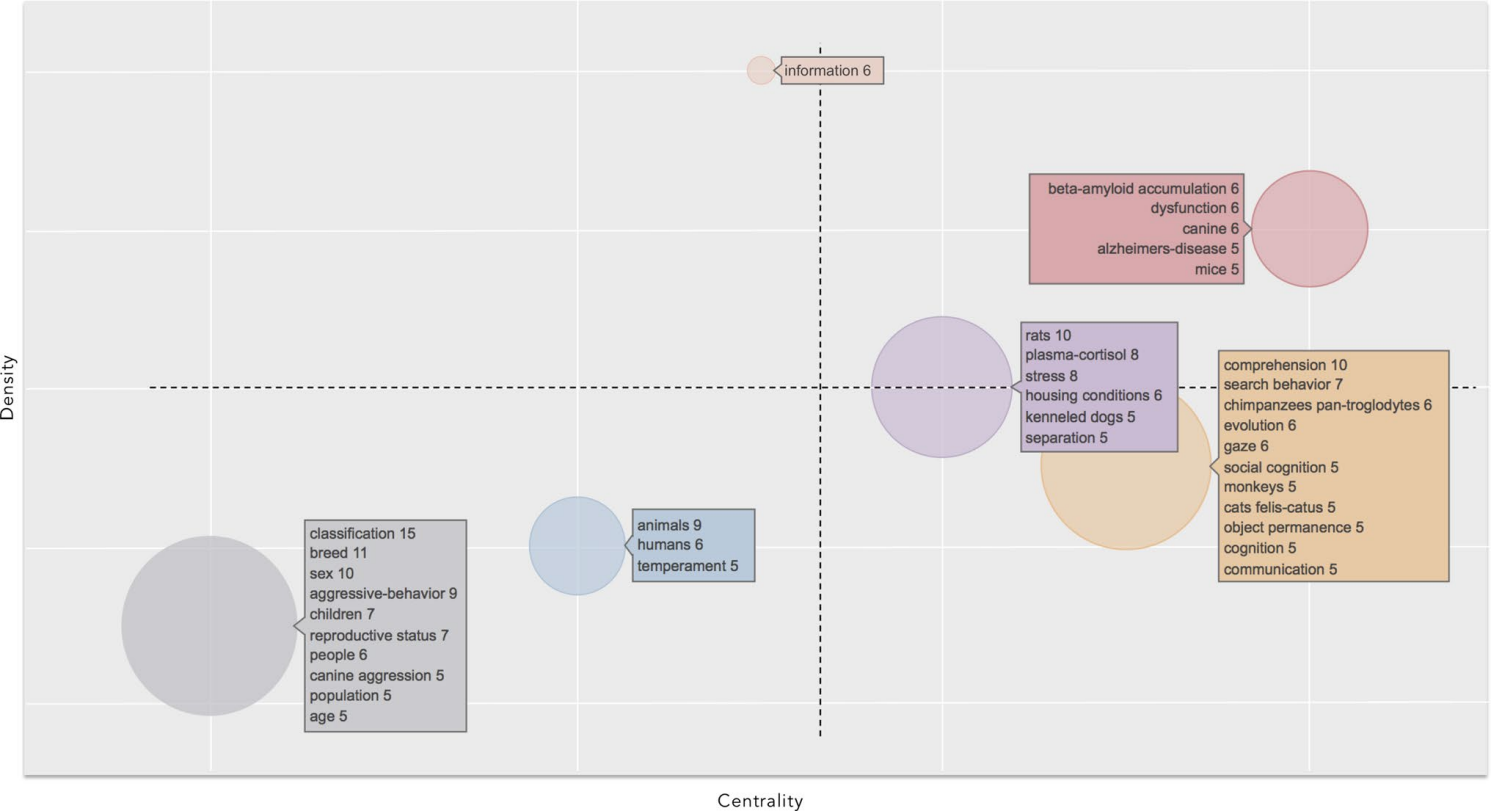
Thematic map showing clusters and the KeyWords Plus from 1993 to 2005 (“earlier analysis”) identified by the co-occurrence network. The X axis represents the centrality (i.e. the degree of interaction of a network cluster in comparison with other clusters) and gives information about the importance of a theme. The Y axis symbolizes the density (i.e. measures the internal strength of a cluster network, and it can be assumed as a measure of the theme’s development). Accordingly, the first quadrant identifies motor themes (i.e. well developed and important themes for the structuring of a research field); in the second quadrant are plotted highly developed and isolated themes (i.e. themes of limited importance for the field); the third quadrant contains emerging or declining themes (i.e. themes weakly developed and marginal); in the fourth quadrant falls basic and transversal themes (i.e. they concerns general topics that are transversal to different research areas of the field)

The analysis of KeyWords Plus in the “earlier analysis” identified six clusters, represented in a thematic map, according to their centrality and density ranking (Fig. 2). A cluster was characterized by high centrality and high density and was positioned in the first quadrant as a motor theme. It included words “beta-amyloid accumulation,” “dysfunction,” and “canine” as the most recurrent terms. A second cluster with good centrality and density identified another well-developed theme, including the KeyWords Plus “rats,” “plasma-cortisol,” and “stress” as the most co-occurring words. Another cluster was in the fourth quadrant, characterized by high centrality and a lower density with respect to the two previous clusters. It was a basic and transversal well-developed theme. The most frequent words were “comprehension,” “search behavior” “chimpanzees pan-troglodytes,” “evolution,” and “gaze.” Two other clusters were characterized by low centrality and low density, which meant they were weakly developed and marginal, and were positioned in the third quadrant. One cluster included the KeyWords Plus “animals,” “humans,” and “temperament”; the other contained, as most frequent KeyWords Plus, “classification,” “breed,” and “sex.” Finally, an isolated theme, with a high density and low centrality, including the only word “information,” was positioned in the second quadrant, which meant that it had limited importance for the field.

**Fig. 3:**
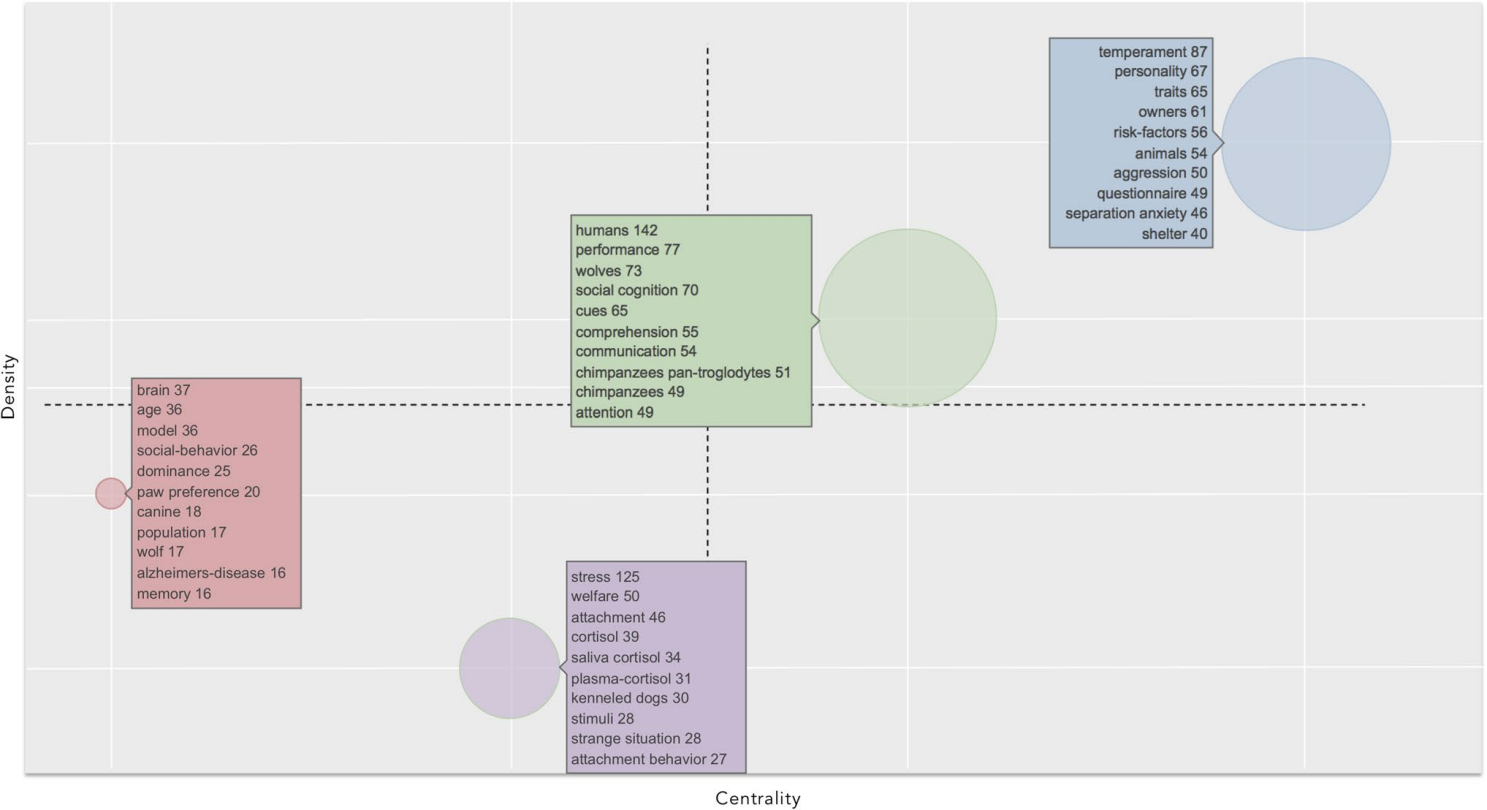
Thematic map showing clusters and the KeyWords Plus from 2006 to 2018 (“recent analysis”) identified by the co-occurrence network. The X axis represents the centrality (i.e. the degree of interaction of a network cluster in comparison with other clusters) and gives information about the importance of a theme. The Y axis symbolizes the density (i.e. measures the internal strength of a cluster network, and it can be assumed as a measure of the theme’s development). Accordingly, the first quadrant identifies Motor themes (i.e. they are well developed and important for the structuring of a research field); in the second quadrant tare plotted highly developed and isolated themes (i.e. they are of limited importance for the field); the third quadrant contains emerging or declining themes (i.e. they are weakly developed and marginal); the fourth quadrant includes basic and transversal themes (i.e. themes concerning general topics transversal to different research areas of the field)

For the period characterizing the “recent analysis”, bibliometrix individuated four clusters, two in the first quadrant and two in the third (Fig. 3). A cluster in the first quadrant was characterized by high centrality and high density and included motor themes in dog cognitive and behavioral studies. The most occurring KeyWords Plus was “temperament,” followed by “personality” and “traits.” The second cluster in the first quadrant had a lower centrality and lower density with respect to the previous cluster and included “humans,” “performance,” and “wolves,” as the most occurring KeyWords Plus. The two clusters in the third quadrant were characterized by low centrality and low density, which meant that they were weakly developed marginal themes. A cluster included “brain,” “age,” “model,” and “social-behavior,” as most common KeyWords Plus, while the other cluster included “stress,” “welfare,” and “attachment.”

### Social structure

**Fig. 4:**
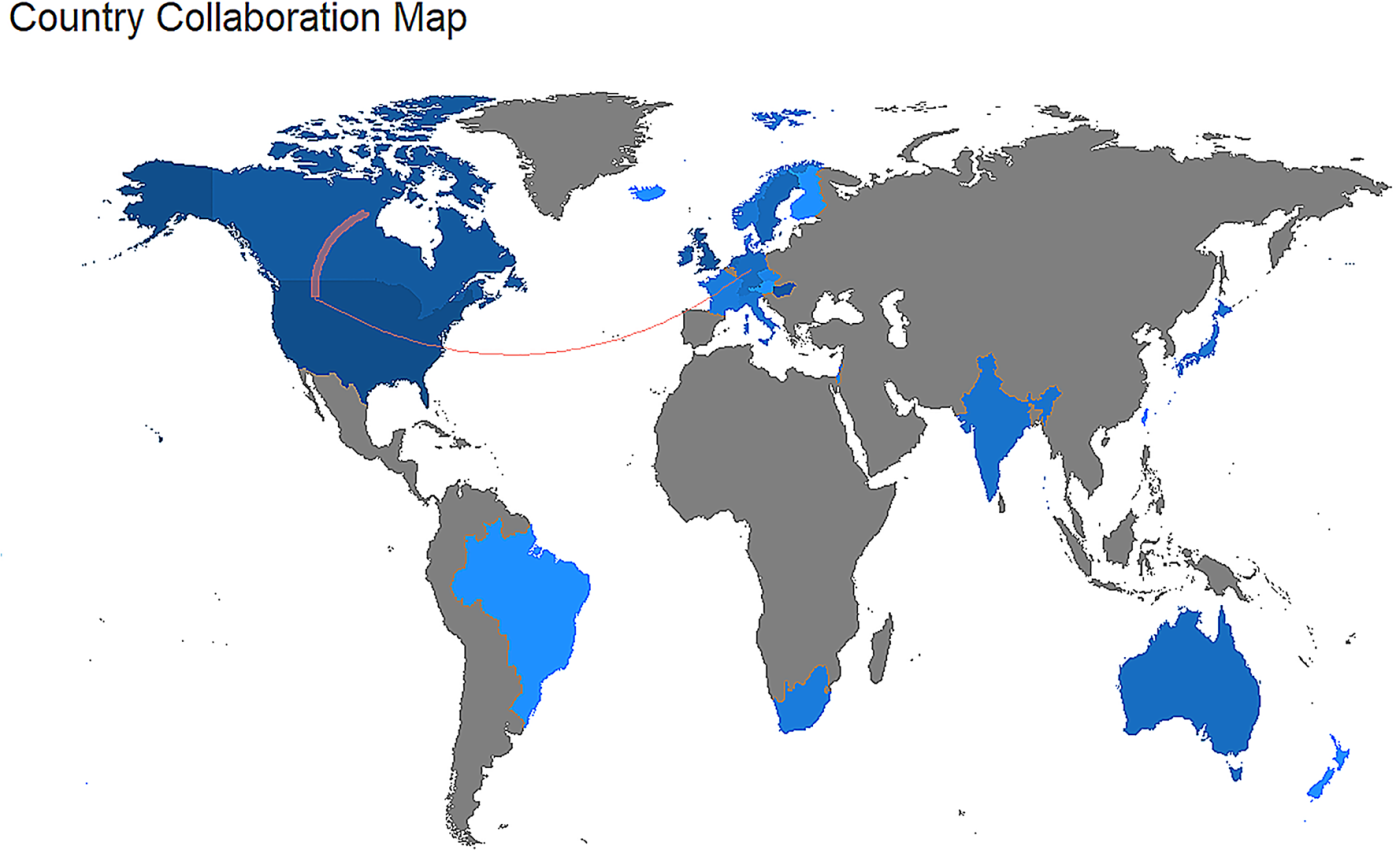
World map showing research collaborations among countries from 1993 to 2005 (“earlier analysis”). Brighter blue color indicates a higher collaboration rate. Countries with less than three shared papers are not shown by connectors

**Fig. 5:**
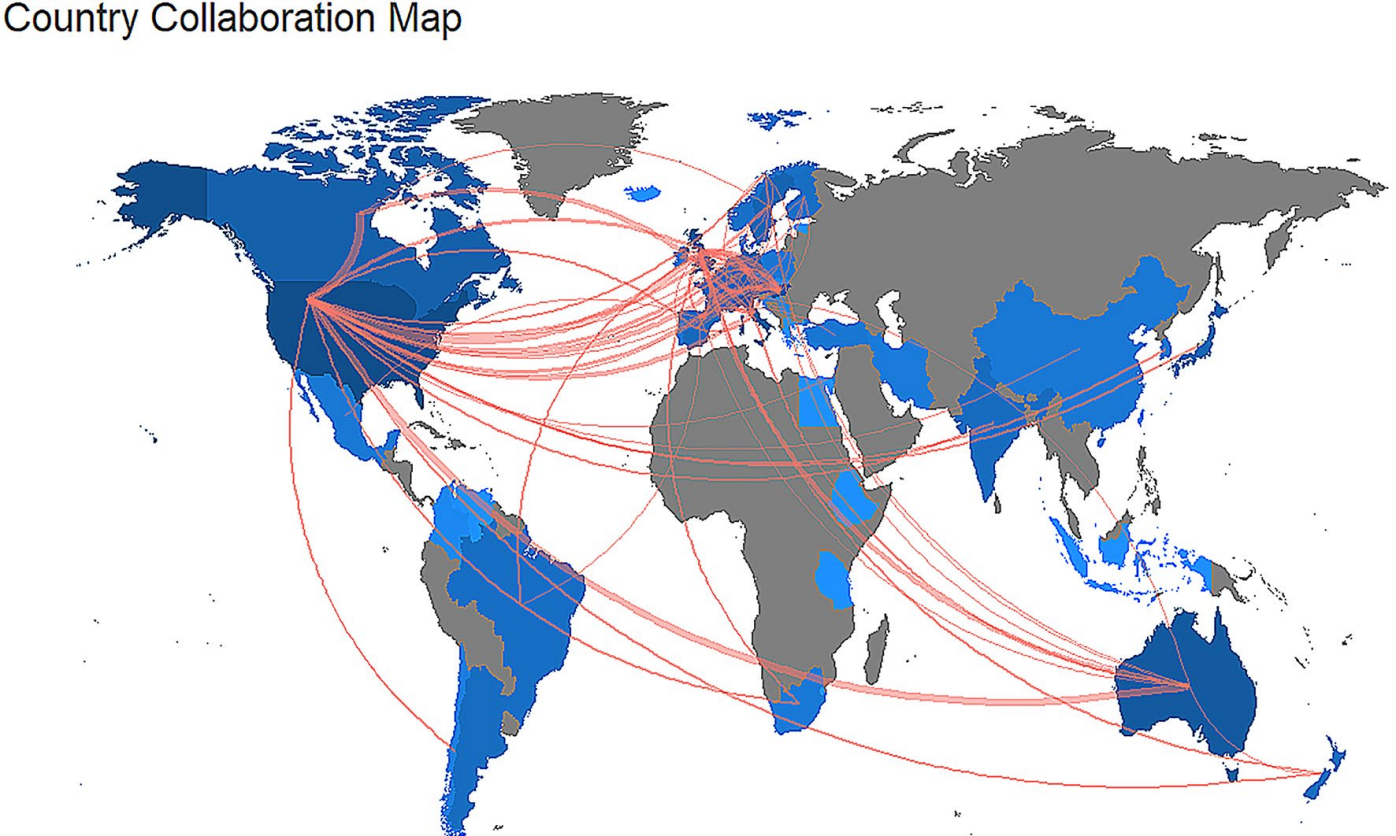
World map showing research collaborations among countries from 2006 to 2018 (“recent analysis”). Brighter blue color indicates a higher collaboration rate. Countries with less than three shared papers are not shown by connectors

The USA and Canada shared the highest number of publications in “earlier analysis”, whereas other countries showed a lower rate of collaboration. In contrast, in the “recent analysis”, a radical increase in the collaboration network is evident (compare Figs. 4 and 5). The USA was again the leading country, sharing most publications with Canada and Australia. Hungary and Austria were at the third position, with several publications in common. It is noteworthy to underlines the radical increase in the collaboration network of the UK. The list of the most collaborative countries can be found in Online Resources 7 and 8.

## Discussion

The first goal of the present paper was to verify whether the dog cognitive and behavioral studies were attracting the interest of scholars more than the studies on cognitive and behavioral sciences in general. It is clear from our data that scientific production for dog cognitive and behavioral studies is substantially more consistent in “recent analysis”, as compared with “earlier analysis”. Furthermore, the growing trend in studies on dogs, starting from 2005, largely exceeds the number of papers in the subject category “Behavioral Sciences,” which we have taken as a reference point for cognitive and behavioral studies in general. Our data provide scientific evidence that in recent times the interest toward the cognitive and behavioral studies on dogs is growing more than the general trend in cognitive and behavioral sciences.

Why the number of studies started to increase around 2005 is difficult to say. The most cited papers in this field came around this period and dealt with dog’ domestication (Hare et al. 2002; Miklósi et al. 2003; Hare and Tomasello 2005). These studies might have provided an important stimulus in raising the interest in the cognitive and behavioral studies on dogs. However, our data show that the interest on the theme related to the dog evolution remained almost unchanged from “earlier analysis” to “recent analysis”; while the studies related to temperament increased considerably (compare Figs. 2 and 3).

Although the total number of papers on dog cognitive and behavioral studies associated with an author’s name has increased, the contribution of a single researcher co-authoring these studies remains almost unchanged, which is in line with the general trend of scientific studies (Fanelli and Larivière 2016). In other words, the number of co-authors per publication increased, but the publication rate per scholar engaged in dog cognitive and behavioral studies did not. This result reflects an increasing trend in the collaboration network from the “earlier analysis” to “recent analysis” (compare Figs. 4 and 5).

Our second goal aimed to understand whether new research themes and/or changes in research themes accompanied the growth of scientific production in dog cognition and behavior starting from 2005. Looking at KeyWords Plus a motor theme in “earlier analysis” was characterized by studies related to aging. Studies in this field used dogs as a model to receive information about the decline of the human brain in elderly people (Bosch et al. 2011). This research theme received limited interest in the “recent analysis” as it showed a decrease in the centrality and density in relationship to the other themes. This means that dogs are less used as models of study for human disease, in comparison with themes aimed to study dogs per se. A similar trend was observed for the research theme related to welfare, which was characterized by good centrality and good density in “earlier analysis”, but it appeared less developed in “recent analysis” in comparison with other themes. Some of the themes maintained a high interest for both the studied periods. Indeed, the theme related to species comparison (evolution) of cognitive and behavioral skills appeared as clusters located almost at the same position in the graph. What deserved more importance from “earlier analysis” to “recent analysis” was the studies related to temperament. This theme appeared as a weakly developed and marginal theme in “earlier analysis”, while it became a motor theme in “recent analysis”. Therefore, this theme was an emerging theme in “earlier analysis”. This was a trend that had already been observed ten years before in another study (Kubinyi et al. 2009). Lastly, a theme including words related to breed, gender, and aggressive behavior classification, appeared as a weakly developed and marginal theme in “earlier analysis”, but it disappeared in “recent analysis”, indicating that it was already a declining theme in “earlier analysis”. It should be underlined that because of the general increase in the cognitive and behavioral studies on dogs in the “recent analysis”, all themes received an increased research effort in recent times but not with the same intensity. Thus, indicating a theme as declining should be interpreted in comparison with other themes in the same period. In this sense, the themes related to aging and welfare were less considered, thus requiring more scientific attention. Overall, both changes in research themes and new research themes contributed to the increased scientific production in dog cognitive and behavioral studies.

About the sources, *Applied Animal Behavior Science* received the highest score by PCA, both in “earlier analysis” and “recent analysis”. However, there was a substantial turnover for other journals, such as *Journal of the American Veterinary Medical Association*, which showed a high score in “earlier analysis”, but not in “earlier analysis”. On the contrary, some sources such as *Animal Cognition* and *Journal of Veterinary Behavior-Clinical Applications and Research* showed a growing trend. Besides, the new sources appearing only in “recent analysis”, such as *PLoS One* and *Scientific Reports*, caused a change in journal ranking, which also reflected as changes in topics (see below). Noteworthy, the ranking is not indicative of the value of a journal since our data do not allow to test for the quality of research reported. Moreover, our analysis refers only a limited field of studies reported in the journals, namely, studies in the cognitive and behavioral science of dogs. Other factors could also affect the ranking, such as the rejection rate, which is different between journals. In recent times, the choice for journal also depends on whether it is open access with a rapid turnover and a larger “reach”. This could be the case with more general journals, like *PLoS One* and *Scientific Reports*.

Among countries, the USA, the UK, and Hungary were the major contributors to the development in dog cognitive and behavioral studies, both in “earlier analysis” and “recent analysis”. While some countries, e.g., Ireland, showed a decline, other countries played a major role, as in the case of Italy and Austria. It should be emphasized that the numbers of research groups differ in the countries. For example, in Hungary and Austria, a single research group is responsible for almost all publications, while several groups in the US and UK contribute to the countries' achievements. The multiple-country publications (MCP), defined as the number of articles including at least a co-author working in a different country with respect to the corresponding author, showed that different countries demonstrated high levels of collaboration activity in “recent analysis”. Indeed, the inter-country collaboration appeared rich in terms of institutions forming large groups of research worldwide. On the other hand, the collaboration net was poorly developed in “earlier analysis”. This outcome is expected since the advances in technology, and the rapid processing and dissemination of scientific information have reduced the barriers of geographic distance and broadened interdisciplinary collaboration.

The data for countries were partially coherent with institutions. Indeed, the Eötvös Loránd University of Hungary provided the maximum contribution to the development of dog cognitive and behavioral sciences, both in “earlier analysis” and “recent analysis”, and the University of Vienna and the University of Milan contributed much in “recent analysis”. Regarding the journals, our analysis does not provide information about the value of a country or an institution and reflects only the studies focused on the cognitive and behavioral science of dogs. It should be remembered that the factors other than the interest in studies on the cognitive and behavioral science of dogs, can affect these outcomes. For instance, the important countries could be driven by research funding, type of institution, or animal ethics regulations. The total number of researchers can affect the number of studies regarding the cognitive and behavioral science of dogs in a country. Some institutions could have had a limited period of activity or started studies on dog cognition and behavior only a while ago. In any case, according to our goal, the present analysis offers a clear picture regarding the most productive countries and affiliations, which is useful to locate where the scientific production on cognition and behavior in dogs is more intense. Moreover, we also provide a map of collaboration, which is useful for several reasons. The ability to debate and share the experience is essential for academic and scientific accomplishment. Constructively, challenging the accepted opinions and ideas is central to their development, and international collaborations help to facilitate this. The collaboration provided a means to professional advancement and increased knowledge (Lukkonen et al. 1992). Beaver and Rosen (1979) concluded that scientific collaboration represents a response to the increasing professionalization of science. Persson et al. (2004) show that citations to articles resulting from international collaborations grow faster than those referring to domestic collaborations. Narin (1991) shows that internationally co-authored articles are more highly cited.

This study has several limitations, which are mainly related to the instrument of bibliometric analysis per second. Indeed, although our selection has limited false-positive, false-negative results are always present in bibliometric research, as it is impossible to generate a perfect and all-encompassing research query.

We included articles only from Web of Science (WoS), and therefore our research cannot cover the entire literature on cognition and behavior in dogs. However, it should be underlined that no scientific database is comprehensive, and each of them, including Scopus or PubMed, has its own strengths and weaknesses (Falagas et al. 2008). Moreover, WoS does not allow electronic access to articles published before 1985. Additionally, many other articles might have been published in not-yet-indexed journals, and therefore they will not be found in any database.

Taking all these limitations into consideration, the number of publications analyzed in this study might not precisely reflect the worldwide research activity on dog cognitive and behavioral studies, but the data presented may still provide significant insight into the evolving trends before and after the increase in dog cognitive and behavioral studies.

In conclusion, it was noted that most of the extant literature on the growing trend in the field of dog cognition and behavioral studies was published after 2000 (Arden et al. 2016), which is in line with our results. Indeed, an increasing trend of publications on dog cognition and behavior is evident in our analysis, which is starting to overlap the cognitive and behavioral studies on other species since 2005. Therefore, it is evident from our data that dogs are attracting the interest of scholars much more than before, and that this effect does not involve, for the same extension, the trend of the cognitive and behavioral research in general. Accordingly, the number of countries and researchers involved in these studies has also increased. In conclusion, it seems that in addition to being the man’s best friend (Miklósi and Topál 2013), the dog is also becoming the scholar’s best friend.

## Electronic supplementary material

Below is the link to the electronic supplementary material.

**Online Resource 1 **Information about sources ordered according to the scores (S) of the principal component analysis from 1993 to 2005 ("earlier analysis"). NP: the number of publications; TC: total citations; h-index: journal’s number of published articles (h), each of which has been cited in other papers at least h time; g-index: the largest number such that the top g articles received at least g^2^citations; m-index: the ratio h/n, where h is the h-index and n the number of years since the first published paper. The first three items are in bold (XLSX 19 KB)

**Online Resource 2 **Information about sources ordered according to the scores (S) of the principal component analysis from 2006 to 2018 ("recent analysis": RA). NP: the number of publications; TC: total citations; h-index: journal’s number of published articles (h), each of which has been cited in other papers at least h time; g-index: the largest number such that the top g articles received at least g^2^citations; m-index: the ratio h/n, where h is the h-index and n the number of years since the first published paper. The first three items are in bold (XLSX 25 KB)

**Online Resource 3 **Information about countries production ordered by the total number of publications by corresponding authors (CNP) from 1993 to 2005 ("earlier analysis"). CFreq: citation frequency according to the publication by the corresponding author; SCP: single country publications (i.e. number of articles in which all authors belong to the same country); MCP: multiple countries publications (i.e. number of articles including at least a co-author working in a different country with respect to the corresponding author); TNP: the number of publications; TC: total citation received; The first three items are in bold (XLSX 11 KB)

**Online Resource 4 **Information about countries production ordered by the total number of publications by corresponding authors (CNP) from 2006 to 2018 ("recent analysis": RA). CFreq: citation frequency according to the publication by the corresponding author; SCP: single-country publications (i.e. number of articles in which all authors belong to the same country); MCP: multiple-country publications (i.e. number of articles including at least a co-author working in a different country with respect to the corresponding author); TNP: number of publications; TC: total citation received; The first three items are in bold (XLSX 13 KB)

**Online Resource 5 **The most productive affiliations from 1993 to 2005 ("earlier analysis": EA). The first three items are in bold (XLSX 11 KB)

**Online Resource 6 **The most productive affiliations from 2006 to 2018 ("recent analysis": RA). The first three items are in bold (XLSX 16 KB)

**Online Resource 7 **Numbers of shared publications between countries in the period 1993–2005 ("earlier analysis": EA) (XLSX 9 KB)

**Online Resource 8 **Numbers of shared publications between countries in the period 2006–2018 ("recent analysis": RA) (XLSX 9 KB)

## Data Availability

The datasets generated and/or analyzed during the current study are available from the corresponding author on request.
